# Protective effect of hydroalcoholic extract of Teucrium polium on diabetes-induced testicular damage and serum testosterone concentration

**Published:** 2017-04-10

**Authors:** Ramin Salimnejad, Ghasem Sazegar, Mohammad Javad Saeedi Borujeni, Seyed Mojtaba Mousavi, Fatemeh Salehi, Fatemeh Ghorbani

**Affiliations:** 1 *Department of Anatomy and Cell Biology, School of Medicine, Mashhad University of Medical Sciences, Mashhad, Iran.*; 2 *Department of Anatomy and Molecular Biology, School of Medicine, Isfahan University of Medical Sciences, Isfahan, Iran.*; 3 *Department of Physiology, School of Medicine, Mashhad University of Medical Sciences, Mashhad, Iran.*; 4 *School of Medicine, Isfahan University of Medical Sciences, Isfahan, Iran.*

**Keywords:** Diabetes mellitus, Oxidative stress, Teucrium polium, Testes

## Abstract

**Background:**

Diabetes has an adverse effect on spermatogenesis by rising oxidative stress.

**Objective:**

The aim of this study was to investigate the effect of Teucrium Polium extract administration on spermatogenesis and testicular structure in diabetic rats induced with Streptozotocin.

**Materials and Methods:**

32 male Wistar rats were randomly divided into four groups (n=8/each): control group, diabetic group received distilled water, and two experimental groups included diabetic rats treated with 50 and 100 mg/body weigh of Teucrium Polium extract for 6 six weeks. After six weeks, the left testis had been removed and the morphometrical study was performed. Blood samples were collected from the ophthalmic veins of the rats and plasma levels of glucose and testosterone hormone were measured afterward.

**Results:**

The reduction in diameters of the seminiferous tubules and thickening of the wall of the seminiferous tubules (p=0.05) were seen in diabetic rats. Also, the degenerative changes in cells arrangement have been observed. Statistical analysis showed the use of Teucrium Polium significantly improved the above disorders in treatment group (100 mg/BW) in contrast to the non treated diabetic group (p=0.05), but no significant difference was seen between the experimental group treated with 50 mg/BW of Teucrium polium and diabetic group (p=0.08). These data also revealed that treatment of diabetic rats with 100 mg/BW of Teucrium Polium extract significantly improves the change in serum glucose (p=0.001) and testosterone (p=0.03).

**Conclusion:**

The results of the present study indicate that diabetes produces degenerative changes in the testis of rats and administration of Teucrium polium reduces complications resulted from diabetes.

## Introduction

One of the most common metabolic disorders is diabetes mellitus (DM) (1, 2). The number of diabetic people in the world is increasing, and it is anticipated that in 2025 approximately 300 million diabetic people will exist in the world (3). Experimental and clinical studies have shown that DM has effects on the male genital system and causes some complications such as decreased testosterone level, seminiferous tubule diameter and sexual instinct and increased abnormal spermatozoa and infertility (4). It is also stated that DM is probably one of the factors that can cause oxidative stress (3-7). Some studies have shown that amplification of an antioxidant system decreases the complications of diabetes (8-10). 

Considering the high prevalence of diabetes and adverse effects on the male reproductive system, it is essential to seek ways to control the disease and its side effects. A long-standing treatment for many diseases is an amplification of medicinal plants and it has been attractive for many physicians. Teucrium polium (T.Polium) is one of the wild-growing flowering species belonging to Teucrium (Laminaceae) genus which has been used for medical purposes in Iran (9, 11, 12). Several studies have reported that T.Polium has hypoglycemic, hypotensive, antibacterial and antipyretic activities (13). Four major flavonoids are yielded from fractionation of T.Polium methanol extract. Flavonoids are secondary plant phenolics which have remarkable antioxidant and chelating effects and are able to scavenge the free radicals produced during lipid peroxidation through donating hydrogen atoms (14, 15). Previous studies have shown that T.polium prevents diabetes-induced oxidative stress (9, 16, 17). 

About the effect of T.Polium on the male reproductive system, there are conflicting with the findings. Wasnaa in 2010 showed that T.Polium aqueous extract significantly increases testicular weight and testosterone levels in the treated group and also increases the number of Leydig's cells, spermatogonia, and spermatozoa in the treated group (18). Al-Ashban *et al* in 2006 showed that chronic treatment with ethanolic extracts of T.Polium causes a significant reduction in weight of the mice testes, an increase in sperm abnormalities and a decrease in blood glucose compared to the control group (19).

In the present study the effect of hydro-alcoholic extracts of T.Polium on diabetes-induced testicular damage and serum testosterone levels have been investigated.

## Materials and methods

**Animals**

In this experimental study, 32 male Wistar rats weighing 250-300 gr and aged approximately 2.5 months old were used. Animals were purchased from the animal house of Mashhad University of Medical Sciences (Spring 2013), and they were maintained in the same place at standard conditions (temperature 22±2^o^C and 12 hr cycle of light/dark). During the study period, they had free access to food and water. 

**Preparation of T. polium extract**

T.Polium was collected from Ferdos, South Khorasan, Iran during the spring. Samples of the plant were identified by the botanist from the Division of Pharmacognosy, Ferdowsi University, Mashhad, Iran (Herbarium No. 152-2016-4), and were dried at room temperature. After the deposition of a voucher specimen, the plants were dried at room temperature. To prepare the hydroalcoholic extract, 50 gr of the dried aerial parts of the plant were chopped and then soaked in ethanol (50%) for 72 hr. After filtering through a paper filter, the extract was dried with the rotary vacuum evaporator. The extract stock was kept in -20^o^C until being used.

**Induction of diabetes**

Diabetes was induced by a single intraperitoneal (I.P) injection of streptozotocin (60 mg/kg). For this purpose, rats were fasted overnight and then were injected. Three days after injection, development of diabetes was confirmed by measuring blood-glucose levels in tail vein blood samples. Rats with blood-glucose levels of 250 mg/dL or higher were considered to be diabetic.

**Study design and experimental groups**

In the present study, 32 male Wistar rats randomly were divided into four groups (n=8/each) according to the experimental protocol: (1) Control, (2) Diabetic, (3) Diabetic-Extract 50 (Dia- Ext 50) and (4) Diabetic- Extract 100 (Dia- Ext 100). Control and diabetic groups received distilled water and treatment groups received a hydro-alcoholic extract of T.Polium (50 and 100 mg/BW) by a gavage once a day for six weeks. At the end of the treatment period, the rats were anesthetized and after perfusion with normal saline and formaldehyde (10%) (Merck, Germany), left testis was removed and then fixed in formaldehyde (10%) at the room temperature. 

After fixation, the testis specimens were dehydrated with an ascending ethanol sequence, cleared with xylene, and embedded in paraffin. Sections (5 μm) were obtained and stained with the hematoxylin and eosin method. Then sections were examined morphometrically with the light microscope (Olympus). For this purpose, in each section we randomly analyzed 50 seminiferous tubules that were round or nearly round, for a total of 200 tubular sections per rat. The inner and outer diameters of the seminiferous tubules and germinal epithelium thickness were measured in these sections. In addition, in this study rats' body weight was measured with a Sartorius digital scale.

**Biochemical assays**

In order to measure the changes in plasma levels of glucose and testosterone hormone, we obtained the blood samples from the rats' ophthalmic veins in three phases, including before the injection, the third week and the sixth week after the start of the study. All the blood sampling phases from rats were performed after 12 hr following by the fasting. Immediately after sampling, blood samples were centrifuged and their serum was stored at -80 until testing. The concentration of glucose was measured by Iran Pars Azmoon kit and by the method of glucose oxidase via the auto analysis device. Plasma levels of testosterone were measured by an ELISA method. 

**Ethical consideration**

The study was approved by the Ethical Committee of Mashhad University of Medical Sciences. 

**Statistical analysis**

All statistical analysis was carried out by using the Statistical Package for the Social Sciences, version 11.5, SPSS Inc, Chicago, Illinois, USA (SPSS software). All data were expressed in mean±SD. The data were analyzed using the One-Way ANOVA followed by Tukey. The results of p≤0.05 were considered as significant. 

## Results

Table I shows the mean of serum glucose level before and during the third week and sixth week of testing. A significant increase has been seen in serum glucose level in the third and sixth wk in the diabetic group in comparison with control group (p=0.001). Additionally, a significant decrease was observed in serum glucose level in the third and sixth wk in the diabetic group that were under treatment with 50 and 100 doses of the hydro-alcoholic extract of T.Polium in contrast to an untreated diabetic group (p=0.001).

**Figure 1:**
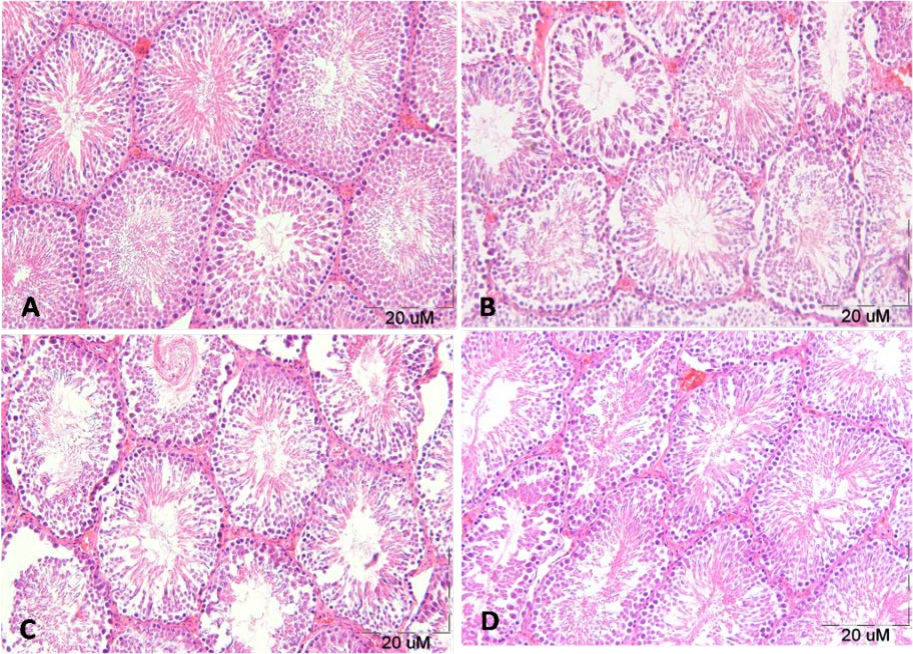
Light microscopy of seminiferous tubules in different groups. Representative photographs of H&E staining in control (A), diabetic (B), Dia- Ext 50(C) and Dia- Ext 100 (D) in rat testis (100X

The seminiferous tubules of the control group had normal germinal epithelium (Figure1-A). Morphological analysis exhibited degenerative changes in the cell's arrangement of seminiferous epithelium in testicular sections of diabetic rats (Figure1-B). Animals treated with T.Polium showed an improvement in testicular sections of diabetic rats (Figure1-C, D).

All the morphometrical data are shown in Table II. Statistical analysis exhibited a significant reduction in the seminiferous epithelium thickness (p=0.01) and diameter of the seminiferous tubules in diabetic rats (p=0.03). These data also showed a significant increase in the diameter of seminiferous tubules (p=0.047) and germinal epithelium thickness (p=0.03) in Dia- Ext 50 when compared to the diabetic group, but no significant difference was seen between Dia- Ext 50 and diabetic groups (p=0.08). 

The result of this study revealed that the body weight of the diabetic group was decreased significantly compared to control group (p=0.05) during the third and sixth week. Treatment of diabetic rats with T.Polium extracts in the third week did not have significant changes in the body weight in contrast to the diabetic group. However, in the sixth week treatments of diabetic rats with an extract of T.Polium showed a significant increase (p=0.05) in the body weight, compared to the diabetic group (Table III). 

**Figure 2:**
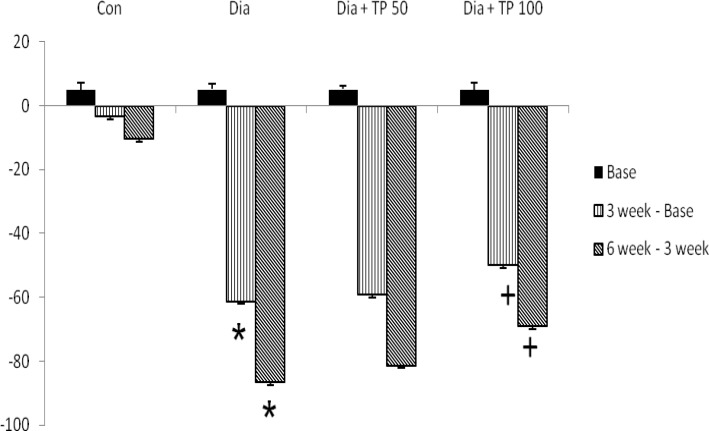
Mean serum testosterone concentrations in pre-treatment (Baseline), third week, and sixth week after the start of the study

Figure 2 shows the changes in percentage of serum testosterone level in the subject group. Serum testosterone level between the subject groups showed that diabetes causes a significant decrease in the level of serum testosterone in the third and sixth week in contrast to control group (p=0.001). Treatment of diabetic rats with 100 dose of T.Polium extracts improved the decrease of the serum testosterone level when compared to the diabetic group (p=0.03), but no significant difference was seen between 50 dose of T.Polium and diabetic group (p=0.12).

**Table I:** Mean serum glucose concentrations in pre-treatment (Baseline), third week, and sixth week after the start of the study

** Glucose level (mg/dl)**	**Baseline**	**Third week**	**Sixth week**
**Groups**			
**Control**	99.78 + 4.13 ---	121.91 + 3.57 ---	124.29 + 4.38 ---
**Diabetic**	449.00 + 6.54 ----	462.55 + 7.50 0.001*	513.45 + 7.33 0.001*
**Dia- Ext 50**	444.37 + 9.02 ---	407.31+ 6.07 0.001+	298.22+ 8.31 0.001+
**Dia- Ext 100**	445.97 + 6.87 ---	343.38 + 4.59 0.001+	194.17 + 4.59 0.001+

All data presented as mean ± S.D

* . P<0.001 compared with control group;

+ P < 0.001 compared with diabetic group. (One-Way ANOVA & Tukey).

**Table II:** Diameter and thickness of the germinal epithelium in all groups

**Parameters**	**Control group**	**Diabetic group**	**Dia- Ext 50 group**	**Dia- Ext 100 group**
**Outer diameter (μm) **	260.12 ± 2.42 ----	251.28 ± 2.71 0.03*	253.47 ± 1.98 0.091	258.48 ± 2.87 0.047+
**Inner diameter (μm) **	211.27 ± 1.26 ---	230.18 ± 1.31 0.03*	228.23 ± 2.26 0.087	224.21 ± 2.01 0.032+
**Thickness of germinal epithelium (μm) **	48.85 ± 1.16 ---	21.1 ± 1.4 0.01*	25.24 ± 0.67 0.1	34.27 ± 0.86 0.03+

All data presented as mean ± S.D

* P<0.03 and P<0.01 compared with control group;

+ P < 0.047 and P<0.03 compared with diabetic group. Also P<0.091 and P<0.1 compared with diabetic group. (One-Way ANOVA & Tukey).

**Table III.** Showing the mean distribution for body weight of all groups before and after study

**Body weight(g)**	**Basic **	**Third week**	**Sixth week**
**Control group**	258.25 + 3.77 ---	268.12 + 5.13 ---	289.00 + 7.21 ---
**Diabetic group**	255.00 + 4.59 ---	246.37 + 5.15 0.05*	224.62 + 4.34 0.05*
**Dia - Ext 50 group**	256.12 + 2.79 ---	249.37 + 1.40 0.09	239.37 + 4.53 0.05+
**Dia - Ext 100 group**	255.32 + 2.09 ---	250.47 + 2.39 0.12	245.17 + 3.43+ 0.05

All data presented as mean ± S.D.

* P<0.05 compared with control group;

+ P < 0.05 compared with diabetic group. Also P<0.09 and P<0.12 compared with diabetic group. (One-Way ANOVA & Tukey).

* (P<0.001) compared with control group; + P<0.03 compared with diabetic group. (One-Way ANOVA & Tukey).

## Discussion

The present study evaluates the protective effect of T.Polium extract against damages inflicted by DM to the male reproductive system. DM caused testicular dysfunctions in male reproductive system and T.Polium extract treatment improved these functional deficits by antioxidant and anti-diabetic roles. The results of this study showed that hydro-alcoholic extract of T.Polium changes serum glucose and testosterone level and also morphological parameters in the diabetic rat testes. 

The results of this study showed that the administration of T.Polium extract decreases blood-glucose levels in the target diabetic rats and this finding correspond to the results of others' studies. The exact mechanism of hypoglycemic activity by T.Polium is not known, but the reasons for blood sugar reduction by T.Polium are increasing in insulin secretion (11). T.Polium performs the process through increasing endogenous insulin secretion, insulin releasing from links, or increasing sensitivity to insulin target tissues (13, 20). In the present study, T.Polium extract probably lowers blood-glucose levels in diabetic rats by a similar mechanism. 

Besides the above-described, we also found a decrease in the plasma testosterone level in diabetic rats. These findings are similar to results of the previous studies; they have reported that during diabetes plasma testosterone levels decrease in the untreated diabetic group (21, 22). 

The decrease in testosterone levels could be associated with the oxidative stress. Oxidative stress due to diabetes increases the ROS that can damage the Leydig cells, and hypothalamic-pituitary axis function leading to decrease the secretion of testosterone and luteinizing hormone (23). Luteinizing hormone has a vital role in spermatogenesis. Luteinizing hormone stimulates the Leydig cells function so that the production of the testosterone hormones (23). The damage of Leydig's cells due to the inhibition of the LH secretion in diabetic rats can be seen from the testosterone level that had been decreased. T.Polium extract improves the testosterone level in the treatment group, because T.Polium contains flavonoids as antioxidants that can counteract free radicals. 

A significant decrease in testosterone level causes degenerative changes in the testis of diabetic rats. On the other hand, the decrease causes damage to the germinal epithelium of seminiferous tubules (24). In this study, histological examination of testis tissue showed that diabetes causes degenerative changes in the seminiferous tubules. Morphometrical examinations of the testes in the diabetic group revealed that the diameter of seminiferous tubules, diameter of lumen and seminiferous epithelium height were significantly decreased, and probably these changes were resulted from spermatogenic cell loss and tubules' disorganization. Additionally, we have shown that some seminiferous tubules had only a single layer of cells attached to the basal lamina. This finding is in line with the previous study documenting the degenerative effects of diabetes on spermatogenesis (3, 23, 25).

Furthermore, regeneration of spermatogenesis was significant in animals treated with the (100 dose) of T.Polium extract. The improvements of decreased diameter of the seminiferous tubules and germinal epithelium thickness were occurred in the treated group, probably due to the antioxidant effect of T.Polium extract that can counteract free radicals (17). 

In fact, there is a positive relationship between the seminiferous tubules diameter and spermatogenesis. In this study, the treatment of diabetic rats with the extract of the T.Polium was an increase in diameter of the seminiferous tubules and spermatogenesis. These findings validate the results of a previous study in which it was shown that the administration of antioxidants reduces the oxidative stress and the amount of testicular damage (2, 10, 20, 26). It is specified that the antioxidant quercetin substance in diabetic rats has ameliorated the complications of diabetes (27). In addition, the administration of melatonin which has antioxidant effects significantly reduced morphological changes in the testes of treated (by melatonin) diabetic rats (28). 

We have also observed that the treatment of diabetic rats with T.Polium extract reduces morphological changes compared to the non-treated group. It can also be due to the antioxidant and antidiabetic effects of T.Polium extract. Using this mechanism, it caused an amelioration of the degenerative changes of the testes tissue. Possible mechanisms involved in the recovery of testicular damage in diabetic rats by T.Polium extract can be expressed as: T.Polium has anti-oxidant effects (flavonoids), decreases blood-glucose levels and increases insulin secretion (9). 

During diabetes, free radicals cause lipid peroxidation and damages to the testes. The antioxidant properties of T.Polium improve the antioxidant system and cause amelioration of the testicular damage. In addition, the regulation of insulin and its direct effects on Sertoli and Leydig's cells have been reported and it is believed that that T.Polium causes an increase in insulin secretion (29). 

It can be stated that T.Polium increases insulin secretion and improves Leydig's cells activity and causes an increase in testosterone secretion by these cells, and the increase of this hormone reduces testicular damage. Furthermore, increases in insulin level can lower blood-glucose level, so with this mechanism testicular damage is also reduced.

## Conclusion

The results of this study validated the antioxidant and anti-diabetic role of the hydroalcoholic extract of T.Polium in improving the testicular damages caused by diabetes. As far as we apperceive, this is the first report about the effect of a hydroalcoholic extract of T.Polium on testicular damage in diabetic rats testis. However, further studies are necessary to confirm these results. Also, it is recommended that in addition to the benefits of T.Polium as a useful material, possible side effects should also be considered.
